# Microbial preference for chlorate over perchlorate under simulated shallow subsurface Mars-like conditions

**DOI:** 10.1038/s41598-024-62346-y

**Published:** 2024-05-21

**Authors:** Florian Carlo Fischer, Dirk Schulze-Makuch, Jacob Heinz

**Affiliations:** 1 Center for Astronomy and Astrophysics, RG Astrobiology, Technische Universität Berlin, Berlin, Germany; 2 GFZ German Research Center for Geosciences, Section Geomicrobiology, Potsdam, Germany; 3 Department of Plankton and Microbial Ecology, Leibniz-Institute of Freshwater Ecology and Inland Fisheries (IGB), Stechlin, Germany

**Keywords:** Astrobiology, Environmental microbiology

## Abstract

The Martian surface and shallow subsurface lacks stable liquid water, yet hygroscopic salts in the regolith may enable the transient formation of liquid brines. This study investigated the combined impact of water scarcity, UV exposure, and regolith depth on microbial survival under Mars-like environmental conditions. Both vegetative cells of *Debaryomyces hansenii* and *Planococcus halocryophilus*, alongside with spores *of Aspergillus niger*, were exposed to an experimental chamber simulating Martian environmental conditions (constant temperatures of about − 11 °C, low pressure of approximately 6 mbar, a CO_2_ atmosphere, and 2 h of daily UV irradiation). We evaluated colony-forming units (CFU) and water content at three different regolith depths before and after exposure periods of 3 and 7 days, respectively. Each organism was tested under three conditions: one without the addition of salts to the regolith, one containing sodium chlorate, and one with sodium perchlorate. Our results reveal that the residual water content after the exposure experiments increased with regolith depth, along with the organism survival rates in chlorate-containing and salt-free samples. The survival rates of the three organisms in perchlorate-containing regolith were consistently lower for all organisms and depths compared to chlorate, with the most significant difference being observed at a depth of 10–12 cm, which corresponds to the depth with the highest residual water content. The postulated reason for this is an increase in the salt concentration at this depth due to the freezing of water, showing that for these organisms, perchlorate brines are more toxic than chlorate brines under the experimental conditions. This underscores the significance of chlorate salts when considering the habitability of Martian environments.

## Introduction

When assessing the habitability of present-day Mars, various environmental factors come into play, among which the availability of water and the radiation environment on and near the surface are crucial considerations^1^. Although there is compelling evidence indicating that Mars once hosted substantial bodies of liquid water, as evidenced by geomorphological features on the surface of Mars^2^, the current conditions render liquid water largely unstable on its surface^3^. However, liquid water could be temporarily stable on and near the surface of present-day Mars in the form of liquid brines^4^, which could be formed either through the contact of salts with water ice, which is supported by experimental evidence^5^, or through a process called deliquescence, in which a hygroscopic salt absorbs water from the atmosphere and dissolves in the absorbed liquid. This is supported by observational evidence of the Phoenix lander, indicating the occurrence of deliquescence on present-day Mars^6^.

Hygroscopic salts are known to be present on Mars, including perchlorate (ClO_4_^−^) salts, which have been detected in quantities of 0.4–0.6 wt% at the Phoenix landing site^7^. Besides perchlorate, the stable intermediate oxychlorine species chlorate (ClO_3_^−^) is likely present on Mars, as indicated by measurements at Gale Crater performed by the Sample Analysis at Mars (SAM) instrument on the Curiosity rover^8,9^ and the detection of chlorate in the Mars meteorite EETA79001^10^. Recent experimental research suggests that under the hyperarid climate and the abundance of iron (hydr)oxide on Mars, chloride oxidation should yield orders of magnitude more chlorate than perchlorate^11^. Additionally, aqueous solutions on Mars may be more likely to be formed by chlorates than perchlorate salts, highlighting their importance for the habitability of Mars^12^. Deposits of (per)chlorate salts or other hygroscopic compounds could be a last means of providing liquid water to the Martian near-surface environment and, therefore, serve as a potential habitat for putative Martian microorganisms^13^.

Besides water availability, radiation on the Martian surface poses a limiting factor to life. The solar UV radiation on the surface of Mars includes wavelengths down to 200 nm, generating DNA damage around three orders of magnitude higher than on the surface of Earth^14^. However, the Martian regolith can attenuate UV radiation so that at a depth range of a few micrometers to millimeters, depending on the particle size of the regolith, the UV radiation is blocked^15–18^. While ionizing radiation on Mars was measured to be 76 mGy/year^19^, it could be shown that doses of that would not be lethal for metabolically active radiotolerant organisms^20^. So, radiation itself may not be a limiting factor to potential microbial life in the shallow subsurface of Mars. However, it is crucial to take into account that ionizing radiation may photolytically generate more reactive oxychlorine species, like chlorites and hypochlorites, from chlorates and perchlorates^21^. A thin regolith layer could also prevent water ice sublimation^22^, thus extending the lifetime of brines and providing a source of water for putative Martian lifeforms, creating possibly favorable habitats in the shallow subsurface.

In this study, we report on how the combined effects of (per)chlorate salts, UV irradiation, water scarcity, and regolith depth affect microbial survival in simulated Mars-like conditions. While other studies report on the impact of perchlorate-containing regolith and regolith UV shielding on the survival of microorganisms^23^, no studies have yet tested the impact of chlorate salts and regolith depths of multiple centimetres. The Mars simulation experiments were conducted in the Mars Environmental Simulation Chamber (MESCH), described in detail by Jensen et al.^24^. The experimental design uniquely enables simultaneous testing of the effects of increased salt stress caused by water freezing at subzero temperatures and sublimation-induced desiccation at various sample depths. This is made possible by the chamber's design which allows to accommodate large sample tubes, enabling the simulation of depths spanning several centimetres. Furthermore, the chamber is outfitted with a locking mechanism that enables the retrieval of samples without disrupting the chamber's internal environmental conditions, so samples can be taken at multiple time points during one experiment. A schematic drawing of the chamber is provided in the method section.

The model organisms used in our experiments were *Debaryomyces hansenii* (a halotolerant yeast), *Planococcus halocryophilus* (a halotolerant and psychrophilic bacterium), and *Aspergillus niger* (a fungal spore former). *D. hansenii* was selected because it exhibits the highest tolerance to NaClO_4_ and NaClO_3_ observed to date^25^, making it an optimal model organism for surviving in chlorate and perchlorate brines*. P. halocryophilus* was isolated from high Arctic permafrost and has demonstrated the ability to grow at a temperature of − 15 °C, the lowest temperature recorded for growth^26^. Additionally, *P. halocryophilus* is capable of growth at perchlorate concentrations of up to 1.1 M NaClO_4_^27^. This makes it a suitable model organism for studying life in cold Martian brines*. A. niger* spores are known for their high tolerance to space and UV-radiation^28^, as well as to desiccation^29^, two significant environmental stressors present on the surface of Mars. Here we report on the survivability of these three organisms under Mars-like surface and subsurface conditions and analysing the impacts of various environmental stress factors including salt stress, UV irradiation, subzero temperatures, and water scarcity.

## Results

Three individual exposure experiments for the three model organisms were conducted in the MESCH. During these experiments, *P. halocryophilus*, *A. niger*, and *D. hansenii* were subjected to the conditions presented in Table 1 for a duration of 3 and 7 days.

**Table 1:** Environmental conditions in the MESCH during the three exposure experiments with *A. niger*, *P. halocryophilus*, and *D. hansenii*.

	*A. niger*			*P. halocryophilus*			*D. hansenii*		
	max	min	avg	max	min	avg	max	min	avg
Temperature (°C)	− 12.2	− 13.7	− 13.2	− 10.1	− 12.0	− 11.0	− 9.5	− 12.0	− 10.8
Pressure (mbar)	6.5	6.0	6.3	7.1	6.3	6.7	6.8	6.2	6.6

The minimum, maximum, and average pressures and temperatures are presented.

The temperature in the chamber fluctuated slightly due to fluctuations in the room temperature, both between and during the three experiments. The pressure increased marginally during the experiments due to the evaporation of water or sublimation of ice from the samples, reaching a maximum increase of 0.8 mbar in the experiments with *P. halocryophilus*. A 150W mercury xenon lamp simulated the Martian UV irradiance levels, and all samples were irradiated on the surface for 2 h per day. The irradiation intensities reaching the interior of the MESCH through the quartz glass window were measured inside the chamber by Jensen et al. at wavelengths of 239 nm, 281 nm, and 365 nm, with respective values of 0.21 W/m^2^, 0.19 W/m^2^, and 0.55 W/m^2^^24^. These values correspond to a total UV dose received at the top of the sample tubes after a 3-day exposure of 4536 J/m^2^ at 239 nm, 4104 J/m^2^ at 281 nm, and 11,880 J/m^2^ at 365 nm. After 7 days of exposure, the accumulated UV doses were calculated to be 10,584 J/m^2^ at 239 nm, 9576 J/m^2^ at 281 nm, and 27,720 J/m^2^ at 365 nm. Calculations by Jensen et al. demonstrated that the intensity of the UV lamp used was about 35 times higher than the average UV flux at 239 nm (averaged over a Martian year and day at 11.6° N) on the surface of Mars^24^. Consequently, the UV radiation doses applied during the experiments—6 h for a 3-day exposure and 14 h for a 7-day exposure—were equivalent to approximately 8.6 and 20 Martian sols, respectively.

In all exposure experiments, three different conditions were tested. Mars regolith simulant was inoculated with cell suspensions of the three model organisms containing either: (1) 0.5 mol/kg NaClO_3_, (2) 0.5 mol/kg NaClO_4_, or (3) no additional salt. These conditions will, in the following sections, be referred to as *NaClO*_*3*_, *NaClO*_*4*_, and *salt-free* samples, respectively.

### Residual water content

**Figure 1:**
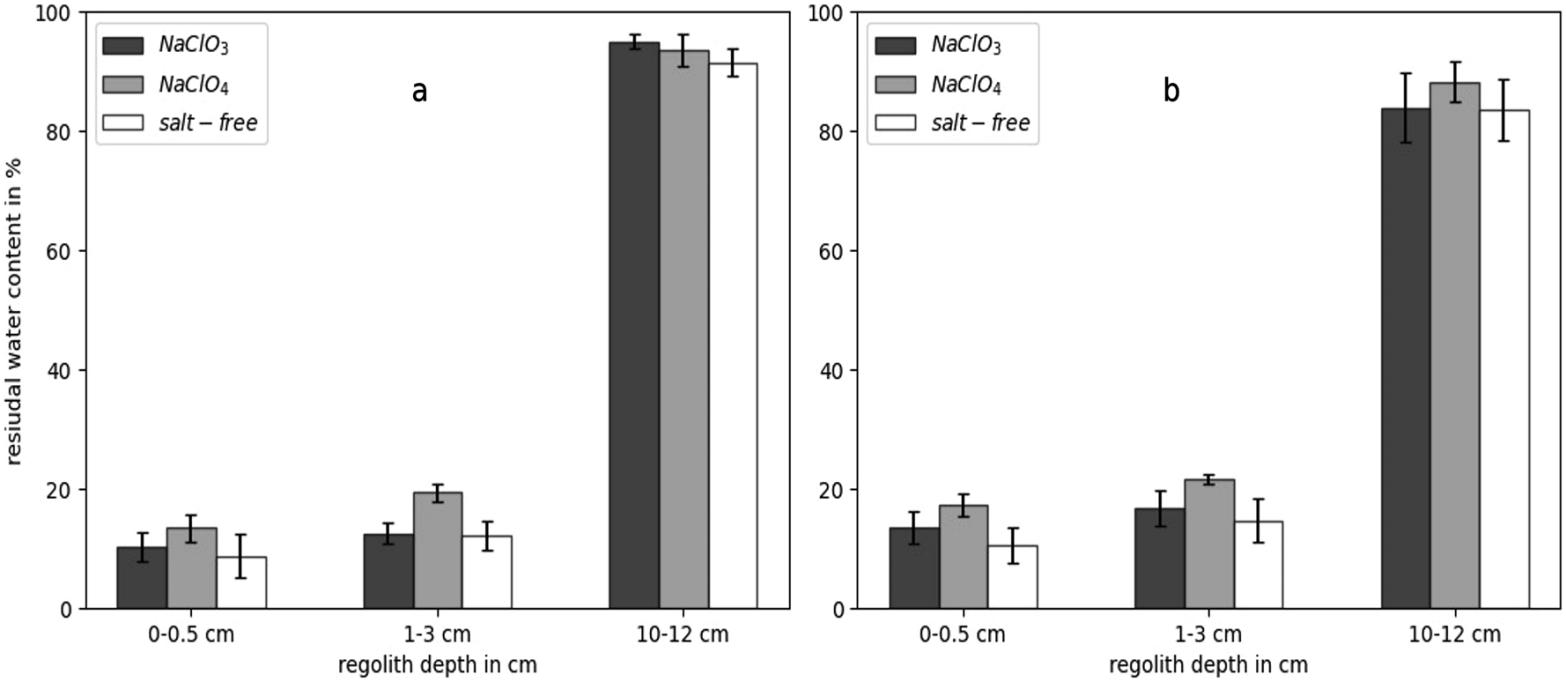
Mean residual water content at the three different tested regolith depths and for the three tested conditions (*salt-free*, *NaClO*_*4*_, *NaClO*_*3*_) after (**a**) 3 and (**b**) 7 days of exposure in the MESCH. The means and standard deviations were calculated from the determined residual water content values in the individual exposure experiments with the three tested organisms (n = 3).

The water content was monitored in all three exposure experiments after 3- and 7-days of exposure in the MESCH. The initial water content generated by the addition of aqueous cell culture to the regolith before exposure was approximately 9 wt% ± 2 wt% (see Supplementary Table S1 online), and the residual water content after exposure was determined at three different depths and for the three experimental conditions (*NaClO*_*3*_, *NaClO*_*4*_, and *salt-free* samples). The calculated mean residual water content is depicted in Fig. 1. The water content was found to increase with depth in all three tested experimental conditions (*NaClO*_*4*_, *NaClO*_*3*_, and *salt-free*). At a depth of 10–12 cm, the residual water content remained above 90% after 3 days and decreased to over 80% after a 7-day exposure. At 0–0.5 cm and 1–3 cm depths, the residual water content was slightly increased after 7 days of exposure compared to the 3-day exposure experiments but never exceeded 25%. Differences in the residual water content among the three conditions (*NaClO*_*4*_, *NaClO*_*3*_, and *salt-free*) were observed to be less than 10% at all depths for both incubation periods (3 and 7 days).

### Survival rates after exposure in the MESCH

**Figure 2:**
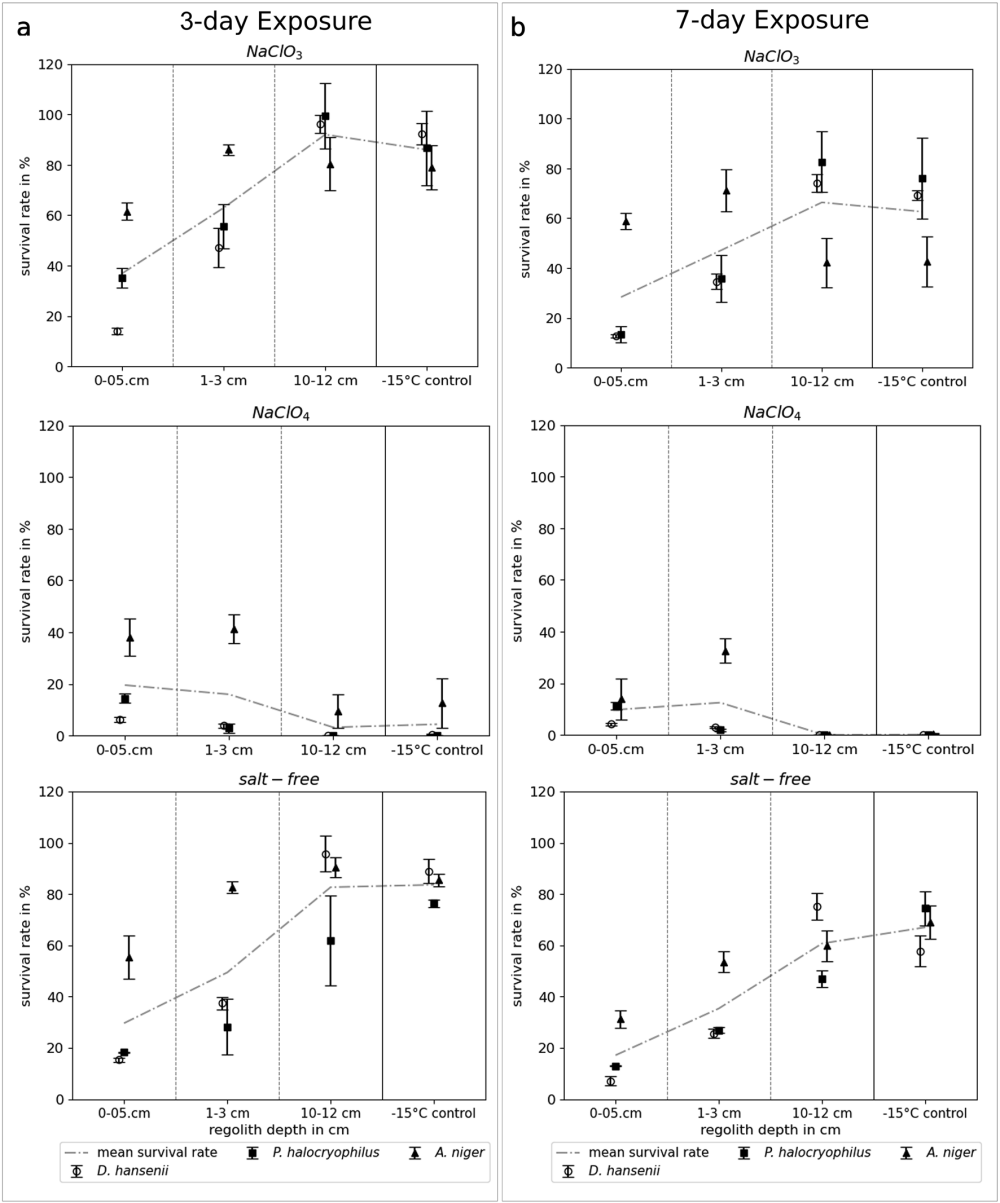
The median of the survival rates of *D. hansenii*, *P. halocryophilus*, *A. niger* (n = 2, SE), and the mean survival rate (dashed line) of the three organisms after (**a**) a 3-day and (**b**) a 7-day exposure in the MESCH. The survival rates are displayed for three sample depths, as well as for the control incubated at − 15 °C in the freezer, in three tested conditions: *NaClO*_*3*_, *NaClO*_*4*_, and *salt-free*.

Three individual exposure experiments were conducted in the MESCH for the three model organisms used—*D. hansenii*, *P. halocryophilus*, and *A. niger*. The survival rates were assessed at both 3 and 7 days of exposure for three depths (0–5 cm, 1–3 cm, and 10–12 cm) and for the three experimental conditions: *NaClO*_*3*_, *NaClO*_*4*_, and *salt-free*. The results are depicted in Fig. 2. All species’ survival rates were substantially higher at a certain depth in the *NaClO*_*3*_ compared to the *NaClO*_*4*_ samples. Notably, the highest differences in survival between *NaClO*_*3*_ and *NaClO*_*4*_ samples were found to be at 10–12 cm sample depth, which coincides with the highest residual water content (Fig. 1). For instance, *P. halocryophilus* displayed a 99.2% survival rate at 10–12 cm in *NaClO*_*3*_ after 3 days, the highest survival rate observed of all organisms, while no survival was detected in *NaClO*_*4*_ samples at the same depth. The survival rates of *P. halocryophilus* and *D. hansenii* in the *NaClO*_*3*_ and *salt-free* samples increased with depth, while the opposite was observed for the *NaClO*_*4*_ samples. This trend was not observed in the experiments with *A. niger*, as *A. niger* spores displayed a higher median survival at 1–3 cm depth compared to 10–12 cm in the *NaClO*_*3*_ samples. Additionally, *A. niger* exhibited a higher survival rate in the *NaClO*_*4*_ samples at 1–3 cm compared to 0–0.5 cm.

*A. niger* generally showed the highest survival rates at 0–5 cm and 1–3 cm compared to the other tested organisms. As opposed to this, at 10–12 cm in the *NaClO*_*3*_ samples, the survival rates of *A. niger* were lower than those of *D. hansenii* and *P. halocryophilus* at the same depth. *A. niger* spores were the only organisms where substantial survival (9% after 3 days) was observed at 10–12 cm in *NaClO*_*4*_. However, after 7 days, no survival was detected in NaClO_4_ at 10–12 cm depth, and only *D. hansenii* displayed a survival rate of 0.2% under these conditions. Furthermore, the differences between the survival rates at 10–12 cm depth and the − 15 °C (freezer control samples) were comparatively small for all tested conditions and organisms.

### Growth at 25 °C

**Figure 3:**
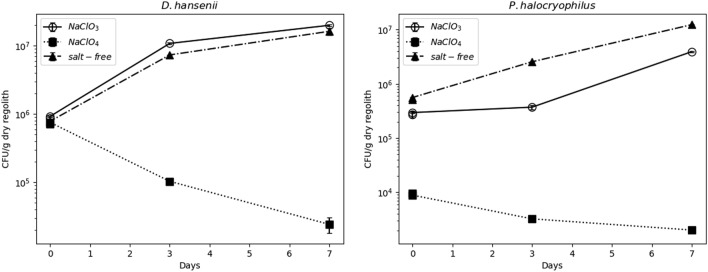
Growth curves of D. hansenii and P. halocryophilus obtained by determining CFU/g dry regolith for control samples, incubated at 25 °C in the incubator under normal atmospheric conditions. CFU/g are drawn against the incubation time in days. The CFU/g were determined in duplicates, and the standard deviation is indicated.

In the control samples incubated for 7 days at 25 °C under normal atmospheric Earth conditions, growth was observed for *D. hansenii* and *P. halocryophilus* in the *NaClO*_*3*_ and *salt-free* samples, while in *NaClO*_*4*_ samples no growth was observed, and a decreasing amount of CFUs was noted (Fig. 3). Additionally, for *P. halocryophilus*, the initial CFU count in *NaClO*_*4*_ was more than an order of magnitude lower than the observed CFUs for *NaClO*_*3*_ and *salt-free* samples due to low cell densities in the NaClO_4_-containing starter culture (see Supplementary Fig. S1 online). As *A. niger* is a filamentous fungus, growth curves could not be obtained through CFU counting.

## Discussion

The performed exposure experiments revealed that all three tested microbial species had a higher survival rate in chlorate (NaClO_3_) compared to perchlorate (NaClO_4_)-enriched regolith simulant under Mars-like conditions. The largest difference in the survival rates between *NaClO*_*3*_ and *NaClO*_*4*_ samples was noted at 10–12 cm depth, coinciding with the highest residual water content. This might be attributed to the emergence of higher salt concentrations at subzero temperatures causing water in the regolith to freeze, leaving behind concentrated salt solutions in both *NaClO*_*3*_ and *NaClO*_*4*_ samples, likely resembling realistic changes in brine concentrations in the Martian shallow subsurface. To estimate the approximate concentration of the resulting salt solution at measured chamber temperatures (Table 1) for regolith depths retaining significant amounts of water (10–12 cm) during the tested time periods, phase diagrams of NaClO_3_ and NaClO_4_ in water were obtained from Hanley et al.^30^ and Hennings et al.^31^, respectively, and data points were then extracted from the diagrams using the Engauge Digitizer (see Supplementary data online). The concentration of NaClO_3_ was determined to be between 3.4 and 2.8 mol/kg, while for NaClO_4_, it ranged from 4 to 3.3 mol/kg, corresponding to temperatures of  −  13.2 °C and − 10.8 °C, respectively. While the determined NaClO_4_ concentration exceeds the highest known microbial NaClO_4_ growth tolerance of 2.5 mol/kg^25^ and is likely too toxic for the tested organisms to show substantial survival over the tested time periods, the determined NaClO_3_ concentrations are within the growth tolerances of organisms such as *D. hansenii* (5.5 mol/kg NaClO_3_)^25^. Our findings contrast with another group’s research using perchlorate-containing regolith, which could not identify adverse effects of perchlorate-containing regolith on the survival of spores of *B. subtilis*^23^. The observed survival of *B. subtilis* by this research group might stem from pre-exposure to desiccation and dry deposition of the perchlorate-containing regolith on the spores, preventing the formation of concentrated perchlorate solutions as observed in our experiments.

Our results suggest that chlorate brines are substantially less toxic than perchlorate brines, also implying a higher potential for life in chlorate brines on Mars. This is underscored by the observation that no growth was observed in the *NaClO*_*4*_ regolith samples incubated at 25 °C, while growth was observed in the *NaClO*_*3*_ samples (Fig. 3). The growth curves furthermore suggest additional stress from the NaClO_4_ regolith mixture, as *P. halocryophilus* and *D. hansenii* grew in 0.5 mol/kg NaClO_4_ in liquid media without additional regolith (Supplementary Figs. S1 and S2 online). Studies with the MGS-1 regolith simulant showed that water-regolith mixtures released elements like S, Ca, Na, and K and that adding perchlorate seemed to enhance the leaching of these elements^32^, possibly elevating their concentrations in the 25 °C *NaClO*_*4*_ samples. This, combined with perchlorate’s toxicity described below, might explain the observed CFU reduction and the lack of growth at 25 °C, while growth was observed in the *NaClO*_*3*_ and *salt-free* samples.

Differences in the microbial tolerance to NaClO_4_ compared to NaClO_3_ were already noted in *D. hansenii*. While *D. hansenii* has the highest tolerance to NaClO_4_ known to date (2.5 mol/kg)^25^, it can tolerate up to 5.5 mol/kg NaClO_3_, which even exceeds its tolerance to NaCl (4.0 mol/kg)^25^. Consistently, a study by Al Soudi et al.^33^ demonstrated that halotolerant bacterial isolates generally exhibited higher tolerance to chlorate than to perchlorate salts. A potential explanation for the observed lower tolerance to NaClO_4_ than to NaClO_3_ is the chaotropic nature of the perchlorate anion, which destabilizes biomacromolecules^34^. Perchlorate-induced stress upregulates protein glycosylation and cell wall remodeling in *D. hansenii*, interpreted as a response to chaotropic destabilization^35^. While chlorate is also chaotropic, it’s considered less so than perchlorate^25^, potentially explaining why NaClO_4_ is more detrimental than NaClO_3_.

Besides great differences in survival rates between *NaClO*_*3*_ and *NaClO*_*4*_ samples, differences were also observed when comparing different sample depths. Survival generally increased with depth in the *NaClO*_*3*_ and *salt-free* samples, except for *A. niger*, which showed a higher median survival at 1–3 cm in *NaClO*_*3*_ samples compared to 10–12 cm. This increase in survival of. *P. halocryophilus* and *D. hansenii* with depth is likely due to greater desiccation at shallower depths. Desiccation can have a detrimental impact on microbes^36^, and as noted in a Mars simulation experiment by Schuerger et al.^23^ it was the second most harmful effect after UV radiation in their experiments. Notably, the survival rates of the *salt-free* samples in the shallower and more desiccated sample depths are comparable to those in the *NaClO*_*3*_ samples, indicating that the increase in NaClO_3_ concentration during the desiccation process does not cause significant additional stress to the organisms on top of the desiccation stress.

Lower survival rates for *A. niger* at a depth of 10–12 cm compared to a depth of 1–3 cm in *NaClO*_*3*_ samples as well as lower survival in *NaClO*_*3*_ samples compared to *salt-free* samples at a depth of 10–12 cm might be linked to the expression of nitrate reductase. *A. niger* is known to possess the *niaD* gene, encoding a nitrate reductase^37^, while neither *D. hansenii*^38^ nor *P. halocryophilus*^39^ possess the ability to reduce nitrate. This enzyme can reduce chlorate to cytotoxic chlorite^40,41^. Among the tested organisms, *A. niger* spores exhibited the highest survival at 0–0.5 cm and 1–3 cm depths. Given that UV radiation and desiccation are more prevalent at these depths than at 10–12 cm and *A. niger'*s known resistance to both^28,29^, this observation aligns with expectations.

For the *NaClO*_*4*_ samples, the decreasing survival rates with increasing depth could be due to a shorter brine stability window at lower depths. Faster desiccation at lower depths prevents brines from persisting for long durations, minimizing the time salt stress is exerted in the organisms.

Previous investigations have reported that perchlorate enhances the bactericidal effects of UV light, likely due to the formation of biologically damaging photoproducts such as hypochlorite and chlorite^42^. This might have increased mortality in the top layer of the 0–0.5 cm *NaClO*_*4*_ samples to a certain extent in our study. However, it is important to note that cells are shielded from UV radiation by several layers of regolith. Although the specific penetration depth of UV radiation in the MGS-1 regolith simulant used in this study is unknown, another study using Mars Mojave Simulant demonstrated that no UV radiation could be detected at a regolith depth of 1 mm^43^. Consequently, assuming a similar penetration depth in MGS-1, samples taken from the depth range of 0–0.5 cm will contain cells both impacted (above 1 mm) and unaffected (below 1 mm) by UV radiation. Therefore, we cannot separate the UV-affected sample fraction from the unaffected fraction, and the detected survival in the sample top layer (0–0.5 cm) is a combination of lethal effects on the surface (UV irradiation and potentially UV-induced hypochlorite and chlorite production) and the protective character of the regolith at depths < 1 mm.

Our study not only measured survival rates but also monitored water content at various depths. Differences in the water retention potential between the upper depths (0–0.5 cm and 1–3 cm) and the 10–12 cm layer were observed under all conditions, demonstrating that regolith layers can decelerate water/brine evaporation. This phenomenon is likely to occur on Mars, potentially stabilizing brines in the shallow subsurface. A study modeling the stability of brines on the surface and in the shallow subsurface suggests that there might exist an optimal subsurface depth where brine evaporation is limited and boiling is suppressed, yet still susceptible to seasonal melting^42^. Chlorate brines at such depths could present a compelling target for future Mars exploration. They are seemingly less toxic, as demonstrated in this work, and, as indicated by recent research^11^, chlorate salts may be even more widespread on Mars than perchlorate salts. Furthermore, it was found that chlorate salts are more likely to form a liquid phase on Mars compared to perchlorate salts^12^.

## Conclusion

This study has demonstrated for the first time that a simulated Mars-like shallow subsurface environment enriched in sodium chlorate (NaClO_3_) is more habitable compared to the analogous sodium perchlorate (NaClO_4_)-enriched environment. This finding, combined with the potential for a more widespread occurrence of chlorate salts on Mars and their higher likelihood of forming liquid brines, calls for further research on this oxychlorine species. To date, most research focused on the toxicity of perchlorate salts, however, according to our results, Martian environments enriched with chlorate salts could be far more habitable than perchlorate-rich environments and should be considered when searching for microbial life on Mars.

## Methods

### Microbial cultures

*Debaromyces hansenii* HUT 7011 (DSM 3428) and *Planococcus halocryophilus* Or1 (DSM 24,743) were obtained from the German Collection of Microorganisms and Cell Culture (Leibniz Institute DSMZ-German, Germany). *D. hansenii* and *P. halocryophilus* were grown aerobically without shaking in their respective DSMZ growth media (DSMZ #90 containing 3% malt extract, and 0.3% soya peptone, and DSMZ #92 containing 3% Tryptic soy broth and 0.3% yeast extract, respectively) at 25 °C. The growth media DSMZ #90 (*D. hansenii*) and DSMZ #92 (*P. halocryophilus*) were supplemented with either 0.5 mol/kg sodium chlorate or 0.5 mol/kg sodium perchlorate or were used without adding salts. Cell growth was monitored by agar plate count and spectrophotometrically measuring the optical density at a wavelength of 600 nm (OD_600_).

### Spore isolation

The *Aspergillus niger* strain N402 was kindly provided by Prof. Dr. Vera Meyer. (Chair of Applied and Molecular Microbiology, Institute of Biotechnology, Technische Universität Berlin, Berlin, Germany), and maintained on DSMZ’#90 agar plates stored at 4 °C. Spores were obtained from 4-day-old cultures grown on DSMZ #90 agar plates at 25 °C. To harvest spores, the agar plates were flooded with autoclaved distilled water, and spores were collected by scraping the plates with a Drigalski spatula. Subsequently, the suspension was filtered through Whatman® qualitative filter paper (Grade 595) to remove any hyphal fragments. Spores were counted using a Neubauer Chamber.

### Mars simulation

**Figure 4:**
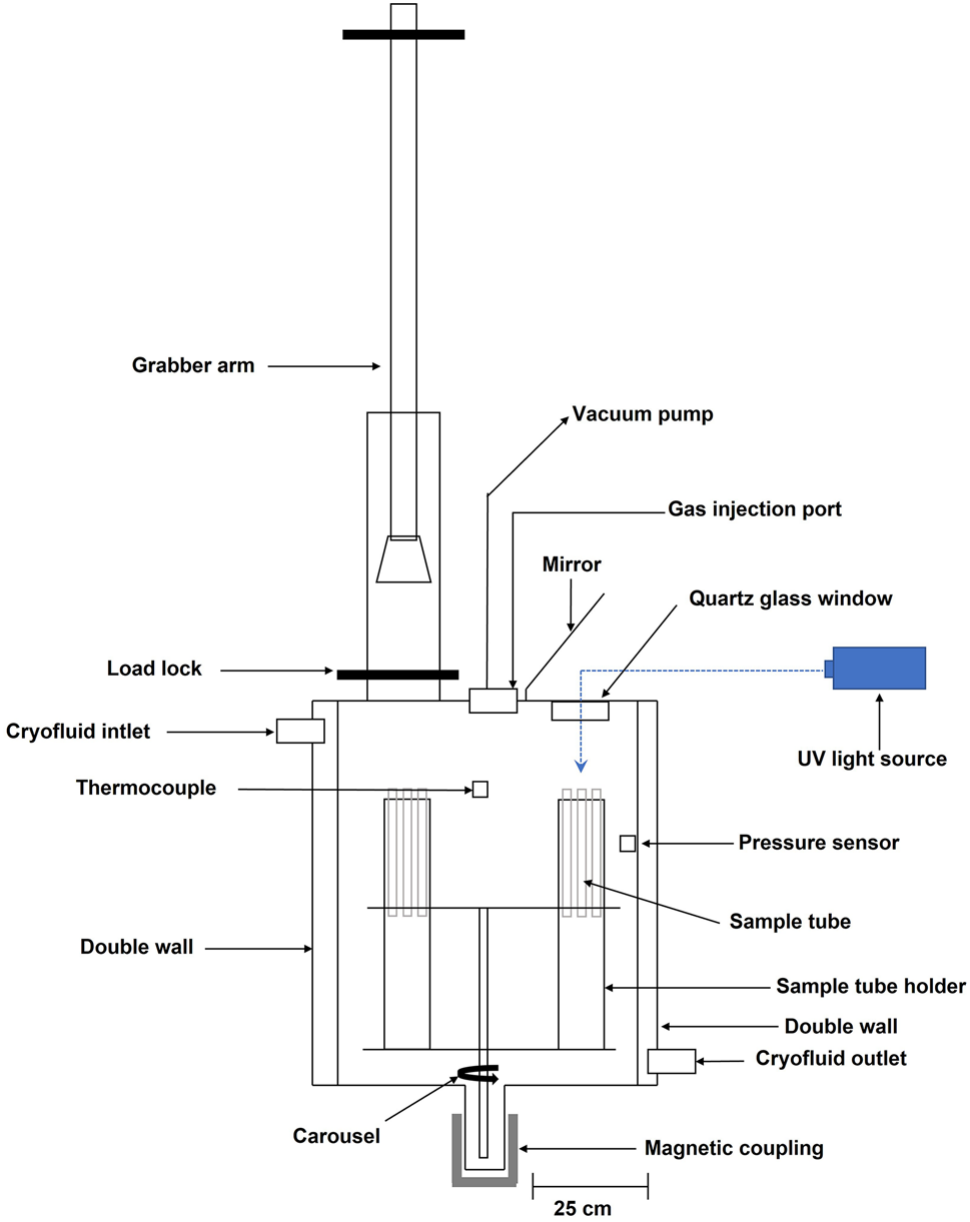
Schematic drawing of the Mars Environmental Simulation Chamber (MESCH) showcases the chamber's structure and the pathway of light from the UV irradiation source to the samples.

Mars simulation experiments were conducted in the Mars Environmental Simulation Chamber (MESCH), described in detail by Jensen et al.^24^. The chamber, illustrated in Fig. 4, comprises a double-walled cylindrical stainless-steel body with internal dimensions of 318 × 396 mm. The double wall is used as a cooling mantle, through which cooling fluid with a constant temperature of − 30 °C is circulated with a cryostat (IKA RC 5 control, IKA-Werke GmbH & CO. KG, Staufen, Germany). The chamber features an airlock system allowing sample retrieval without disturbing the environmental conditions. A connected vacuum pump and CO_2_ gas flask enable air evacuation and the introduction of CO_2_, allowing to establish a Mars-like atmosphere of approximately 6–7 mbar CO_2_. UV radiation was generated using a 150 w Xenon mercury lamp (Hamamatsu Photonics L2482, Hamamatsu Photonics Systems, Shizuoka, Japan), and the samples were irradiated through a quartz glass window on top of the chamber. Samples can be rotated to be positioned under this window with the help of a remotely controlled stepper motor**.** Temperature and pressure in the chamber were measured every 30 min by a thermocouple (Honeywell, Minneapolis, USA) and pressure sensor (Pfeiffer PCR-260, Pfeiffer Vacuum GmbH, Asslar, Germany).

### Exposure experiments

Cultivation of *D. hansenii* and *P. halocryophilus* followed the procedure outlined before. The cell cultures of *D. hansenii* and *P. halocryophilus* were harvested in the late exponential growth phase and diluted tenfold using their respective growth media supplemented with 0.5 mol/kg NaClO_3_, 0.5 mol/kg NaClO_4_, or no added salt. The dilution step was omitted for the *P. halocryophilus* culture grown in 0.5 mol/kg NaClO_4_ due to low cell densities observed in this culture. *A. niger* spores were harvested as described above. The resulting spore solution was divided into three equal portions and centrifuged at 3000 g for 5 min, and the resulting pellets were resuspended in DSMZ #90 growth media containing either 0.5 mol/kg NaClO_3_, 0.5 mol/kg NaClO_4_, or no added salt.

**Figure 5:**
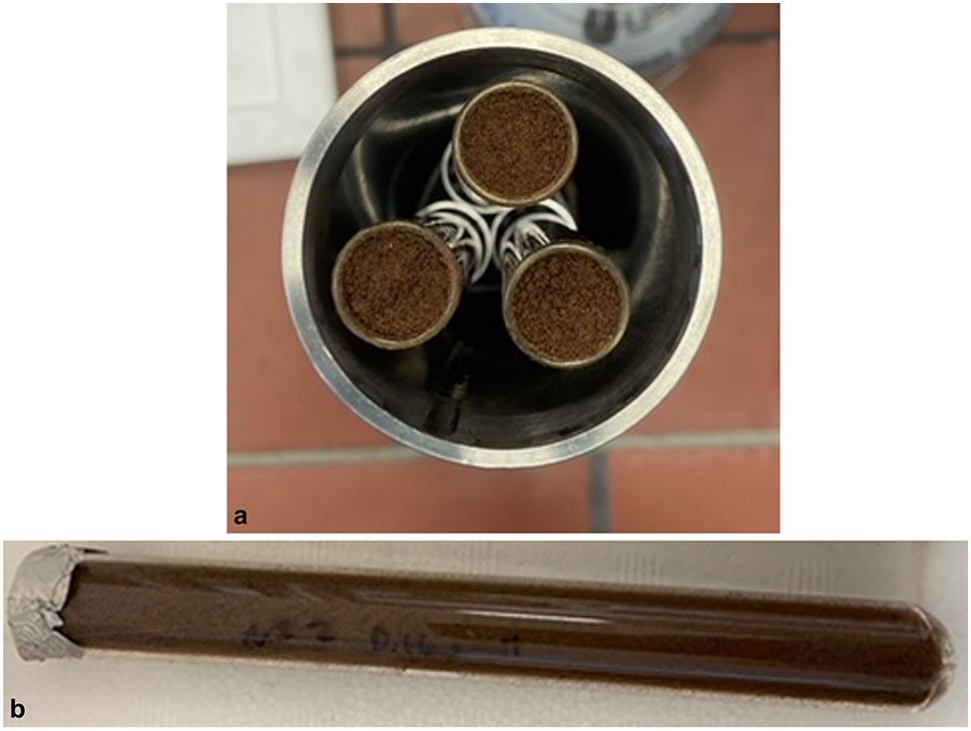
Images of the configuration of the exposure experiments showing (**a**) three sample tubes in the stainless steel sample tube holder and (**b**) an individual sample tube.

Thirty grams of Mars Global Simulant (MGS-1, Exolith Lab, Orlando, USA) were homogenously inoculated with 3 g of these spore or cell solutions, containing either 0.5 mol/kg NaClO_3_, 0.5 mol/kg NaClO_4_, or no additional salt. This was achieved by thoroughly mixing the solution and the regolith with a sterilized spatula until the regolith was uniformly moist. The inoculated regolith was then transferred into glass reaction tubes with a depth of 15 cm. These tubes were subsequently placed within a steel sample holder tube (3 sample tubes each, Fig. 5) and transferred to the pre-cooled MESCH set at approximately − 10 to − 13 °C. Using 3 g of a 0.5 mol/kg sodium perchlorate culture solution results in approximately 0.184 g of sodium perchlorate added to 30 g of regolith, equivalent to about 0.15 g of ClO_4_^−^ per 30 g of regolith. This corresponds to a calculated perchlorate content in the dry regolith (not considering the varying residual water content) of 0.5 wt%. This concentration aligns with Phoenix rover measurements, which recorded ClO_4_^−^ concentrations between 0.4 and 0.6 wt% ClO_4_^–^^7^.

Control samples were prepared by placing 1 g of inoculated regolith in 15 ml reaction tubes and storing them with sealed lids in either a freezer (− 15 °C) or a 25 °C incubator under normal atmospheric conditions and without UV irradiation. The initial cell or spore count (CFU/g regolith) and the initial water contents were determined according to the methodology outlined below.

Once all samples were positioned within the MESCH, the vacuum pump was activated to evacuate the chamber until a pressure of approximately 1 mbar was attained. The chamber was flooded with CO_2_ to a pressure of around 1 bar. This process was repeated three times, followed by a gradual adjustment of the pressure to a range of 6–7 mbar by slowly introducing CO_2_ into the MESCH. Following an exposure duration of 3 days, one steel tube containing three glass reaction tubes was removed from the chamber using the airlock system without disrupting the environmental conditions inside. After a total exposure period of 7 days, the steel tube containing the remaining samples was retrieved from the chamber. This procedure was executed for all three organisms under investigation.

### Water content determination

The water content of the exposure samples was determined in duplicates by weighing samples of the regolith taken before and after (at different depths) the exposure in the MESCH chamber.

The samples were weighed before and after desiccation in an oven at 40 °C, and the water content (*w)* as well as the residual water content (*w*_*res*_*)* was calculated by the difference in weight as illustrated in Eq. (1). The masses *m*_*wet*_ and *m*_*dry*_ correspond to the masses before and after the desiccation in the oven, respectively. In the equation,* w*_*start*_ represents the water content before, and *w*_*end*_ is the water content after the exposure.

1 $$w=\frac{{{m}_{wet-}m}_{dry}}{{m}_{wet}}\times 100\% ; {w}_{res }=\frac{{w}_{end}}{{w}_{start}}$$

### CFU/g determination and survival rate calculation

Survival rate was determined by assessing the CFU/g of regolith before, during (3-day exposure), and after (7-day exposure) the exposure experiments. Approximately 0.5 g of regolith from depths of 0–0.5 cm, 1–3 cm, and 10–12 cm were aseptically collected using a sterilized stainless-steel spoon. This involved carefully removing layers of regolith until the desired depth was reached. The regolith from the corresponding depth range was transferred to a sterile petri dish, homogenously mixed with a sterile spatula, and then transferred into 15 ml reaction tubes and weighed. One g of Phosphate Buffered Saline (PBS) per 0.1 g of regolith was added. This procedure was performed in duplicates for every depth and for the initial inoculated regolith samples before exposure. Subsequently, the solution was serially diluted with PBS and plated on the respective agar plates for the organisms. CFUs were counted after 3 days (*A. niger* and *D. hansenii*) or after 5 days (*P. halocryophilus*) of colony growth.

Using sufficient approximation, it is considered that 1 g of PBS is equivalent to 1 ml of PBS, and similarly, 1 g of the regolith/PBS mixture is equivalent to 1 ml of this solution. Consequently, when 10 g of PBS are utilized to suspend one gram of regolith to accurately represent the CFU/g of wet regolith, the measured CFU/ml are multiplied by 11, reflecting the total mass of the mixture in this approximation. Using the determined water content ($w$), the CFU/g dry regolith were determined, as shown in Eq. (2).

2 $$\frac{CFU}{{g \;\;dry \;\;regolith}} = \frac{{\frac{CFU}{{ml}} \times 11}}{{\left( {1 - \frac{w}{100}} \right)}}$$

The survival rate (*S*) is determined by calculating the ratio of the initial CFU/g dry regolith (*CFU/g dry regolith start*) to the value after the exposure periods (*CFU/g dry regolith end*), as demonstrated in Eq. (3).

3 $$S = { }\frac{{\frac{CFU}{{g{ }\;\;dry{ }\;\;regolith}}_{start} }}{{\frac{CFU}{{g{ }\;\;dry{ }\;\;regoltih}}_{end} }} \times 100{\text{\% }}$$

The CFU/g of dry regolith (CFU/g) was determined in technical duplicates for all samples and depths, resulting in two initial CFU/g values and two CFU/g values after exposure. The survival rate was calculated by taking the ratio between the median of the initial CFU/g values and the median of the CFU/g values after exposure. The uncertainty (standard error) in the survival rate (dS) was calculated using Eq. (4):

4 $$dS= \left(\sqrt{{\left(\frac{d{CFU}_{inital}}{{CFU}_{inital}}\right)}^{2}+ {\left(\frac{d{CFU}_{end}}{{CFU}_{end}}\right)}^{2} }\right)\times S$$

Here in, *dCFU*_*initial*_ represents the difference between the duplicates of the initial CFU/g values and *dCFU*_*end*_, the difference between the technical duplicates of the CFU/g values after the exposure. *CFU*_*inita*l_ and *CFU*_*end*_ represent the median of the initial CFU/g value and the CFU/g value after the exposure, respectively.

### Supplementary Information

Supplementary Information 1.

Supplementary Information 2.

## Data Availability

Data is provided within the manuscript or supplementary information files. For numerical data presented in the figures, data is also available in tabular from the contact author Jacob Heinz upon request (heinz@tu-berlin.de).
