# Supplementary material for: Microbial preference for chlorate over perchlorate under simulated shallow subsurface Mars-like conditions
Source: Sci Rep. 2024 May 21;14:11537. doi: 10.1038/s41598-024-62346-y (PMC11109124; doi:10.1038/s41598-024-62346-y)
Supplement: Supplementary file 1 — Supplementary Information 1. [file 41598_2024_62346_MOESM1_ESM.pdf]

Supplementary Information:

Chlorate increases microbial survivability in a simulated shallow subsurface Mars-like environment

Author List and Affiliations:

FISCHER, FLORIAN CARLO[1]; Schulze-Makuch, Dirk[1,2,3]; Heinz, Jacob[1*]

[1] Center for Astronomy and Astrophysics, RG Astrobiology, Technische Universität Berlin, Berlin, Germany

[2] GFZ German Research Center for Geosciences, Section Geomicrobiology, Potsdam, Germany

[3] Department of Plankton and Microbial Ecology, Leibniz-Institute of Freshwater Ecology and Inland Fisheries (IGB), Stechlin, Germany.

Contact Information:

Fischer, Florian Carlo: florian.fischer@tu-berlin.de

Schulze-Makuch, Dirk: schulze-makuch@tu-berlin.de

Heinz, Jacob: heinz@tu-berlin.de

.

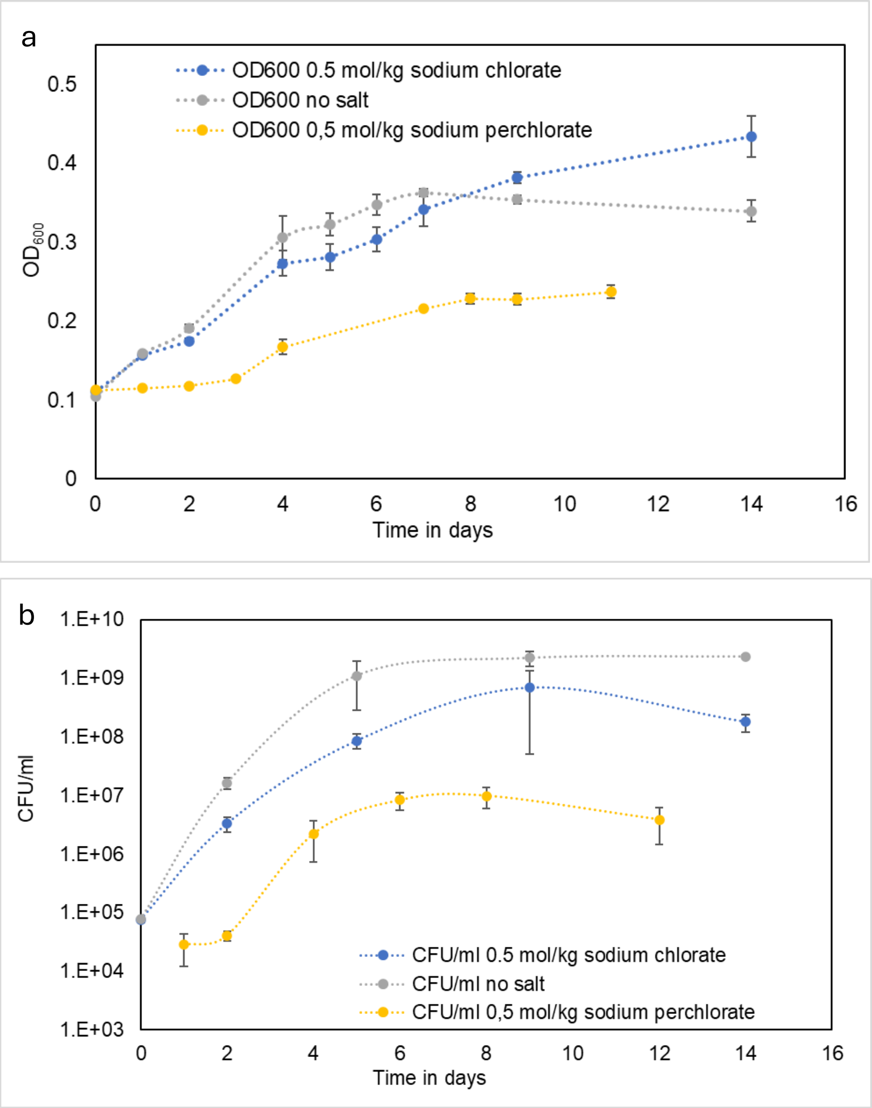

Figure S1: Growth curves of *P. halocryophilus* in complex growth medium with or without adding 0.5 mol/kg sodium (per-)chlorate. (a) Counted cell densities in CFU/ml over the course of cultivation. (b) OD_{600} drawn against the cultivation time.

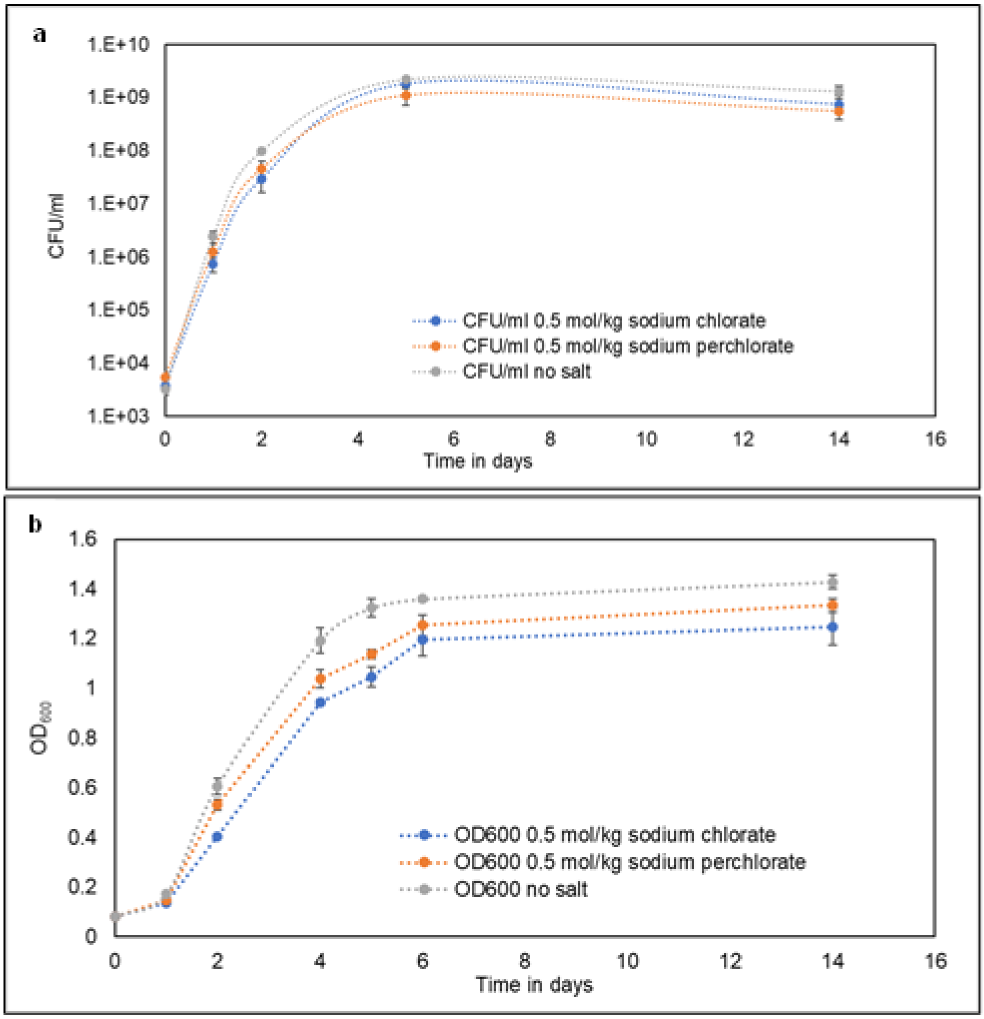

Figure S2: Growth curves of *D. hansenii* in complex growth medium either with or without the addition of 0.5 mol/kg sodium (per-)chlorate. (**a**) Counted cell densities in CFU/ml drawn against the time in days. (**b**) OD_{600} over the course of 15 days of cultivation.

Table S1: The determined water content at the start of the exposure experiments. Water content data is shown for the 3- and 7- day samples and for all three tested conditions and organisms. Water content was determined in duplicates for each condition (n=2).

	A. niger			***P. halocryophilus***			***D. hansenii***		
**3-Days samples**	***$NaClO_3$***	***$NaClO_4$***	***salt-free***	***$NaClO_3$***	***$NaClO_4$***	***salt-free***	***$NaClO_3$***	***$NaClO_4$***	***salt-free***
start water content N1 [%]	9.13	9.15	8.91	8.89	8.98	9.87	8.60	10.34	9.05
start water content N2 [%]	9.70	9.95	9.39	9.59	9.87	9.41	9.79	10.84	9.99
**7-Days samples**	***$NaClO_3$***	***$NaClO_4$***	***salt-free***	***$NaClO_3$***	***$NaClO_4$***	***salt-free***	***$NaClO_3$***	***$NaClO_4$***	***salt-free***
start water content N1 [%]	9.03	8.97	8.87	7.29	7.91	7.17	8.40	9.27	9.00
start water content N2 [%]	10.43	9.58	9.30	7.94	8.20	9.32	10.21	10.44	9.39
